# Attitudes, Beliefs, and Willingness Toward the Use of mHealth Tools for Medication Adherence in the Florida mHealth Adherence Project for People Living With HIV (FL-mAPP): Pilot Questionnaire Study

**DOI:** 10.2196/12900

**Published:** 2019-07-03

**Authors:** Jamie P Morano, Kevin Clauson, Zhi Zhou, César G Escobar-Viera, Spencer Lieb, Irene K Chen, David Kirk, Willie M Carter, Michael Ruppal, Robert L Cook

**Affiliations:** 1 Department of Infectious Diseases and International Medicine Morsani College of Medicine University of South Florida Tampa, FL United States; 2 Clinical Research Unit University of South Florida Florida Department of Health - Hillsborough Tampa, FL United States; 3 Department of Pharmacy Practice College of Pharmacy and Health Sciences Lipscomb University Nashville, TN United States; 4 Department of Epidemiology College of Public Health and Health Professions and College of Medicine University of Florida Gainesville, FL United States; 5 Southern Alcohol HIV Research Consortium Center for Translational HIV Research University of Florida Gainesville, FL United States; 6 Center for Research on Media, Technology, and Health - Health Policy Institute University of Pittsburgh Pittsburgh, PA United States; 7 The AIDS Institute / Florida Consortium for HIV/AIDS Research Tampa, FL United States; 8 Immunology Clinical Research Unit Florida Department of Health - Orange County Orlando, FL United States

**Keywords:** mHealth, mobile phone app, app, HIV, antiretroviral therapy adherence, persons living with HIV, HIV care continuum, digital health, medication adherence, mobile health

## Abstract

**Background:**

Antiretroviral (ART) adherence among people living with HIV (PLWH) continues to be a challenge despite advances in HIV prevention and treatment. Mobile health (mHealth) interventions are increasingly deployed as tools for ART adherence. However, little is known about the uptake and attitudes toward commercially available, biprogrammatic mobile apps (ie, designed for both smartphone and short message service [SMS] messaging) among demographically diverse PLWH.

**Objectives:**

The Florida mHealth Adherence Project for PLWH (FL-mAPP) is an innovative pilot study that aimed to determine the acceptability of a commercially available, biprogrammatic mHealth intervention platform to ensure medication adherence and gauge the current attitudes of PLWH toward current and future mHealth apps.

**Methods:**

A predeveloped, commercially available, biprogrammatic mHealth platform (Care4Today Mobile Health Manager, Johnson & Johnson, New Brunswick, NJ) was deployed, with self-reported ART adherence recorded in the app and paper survey at both short term (30-day) or long-term (90-day) follow-ups. Consented participants completed baseline surveys on sociodemographics and attitudes, beliefs, and willingness toward the use of mHealth interventions for HIV care using a 5-point Likert scale. Chi-square tests and multivariate logistic regression analyses identified correlations with successful uptake of the mHealth platform.

**Results:**

Among 132 PLWH, 66% (n=87) initially agreed to use the mHealth platform, of which 54% (n=47) successfully connected to the platform. Of the 87 agreeing to use the mHealth platform, we found an approximate 2:1 ratio of persons agreeing to try the smartphone app (n=59) versus the SMS text messages (n=28). Factors correlating with mHealth uptake were above high school level education (adjusted odds ratio 2.65; *P*=.05), confidence that a clinical staff member would assist with mHealth app use (adjusted odds ratio 2.92, *P*=.048), belief that PLWH would use such an mHealth app (adjusted odds ratio 2.89; *P*=.02), and ownership of a smartphone in contrast to a “flip-phone” model (adjusted odds ratio 2.80; *P*=.05). Of the sample, 70.2% (n=92) reported daily interest in receiving medication adherence reminders via an app (80.4% users versus 64.7% nonusers), although not significantly different among the user groups (*P*=.06). In addition, 34.8% (n=16) of mHealth users reported a theoretical “daily” interest and 68.2% (n=58) of non-mHealth users reported no interest in using an mHealth app for potentially tracking alcohol or drug intake (*P*=.002).

**Conclusions:**

This commercially available, biprogrammatic mHealth platform showed feasibility and efficacy for enhanced ART and medication adherence within public health clinics and successfully included older age groups. Successful use of the platform among demographically diverse PLWH is important for HIV implementation science and promising for uptake on a larger scale.

## Introduction

Antiretroviral therapy (ART) adherence is a recognized cornerstone in suppressing HIV viral load (VL) and thus achieving positive health outcomes in people living with HIV (PLWH) and their partners. As per the 2020 Joint United Nations Programme on HIV/AIDS target, 90% of all people with diagnosed HIV infection will receive sustained antiretroviral therapy (ART) and 90% of all people receiving antiretroviral therapy will have viral suppression [1]. As PLWH successfully complete longer lifespans, it becomes critical to optimize medication adherence for cardiovascular disease, cancer, diabetes mellitus, and hypertension in addition to overall health outcomes for PLWH, especially with the increasing overall pill burden and increasing requirement for care management by multispecialty health care providers.

In the United States, 70% of PLWH were estimated to have HIV VL suppression (defined as <200 copies/mL) when linked to care, as per the Centers for Disease Control and Prevention Medical Monitoring Project 2016 report [2]. ART adherence continues to be a challenge in the HIV treatment cascade, especially among those with poor social support, increased stigma, comorbid alcohol and substance use, and mental health challenges, all of which often are exacerbated by the challenges of poverty, housing and food instability, and inadequate transportation [3,4]. Optimal ART adherence is needed to help reduce the number of new HIV infections, improve health outcomes, and reduce health disparities [5,6]. Although much progress has been made in HIV prevention in the United States [7], and the ability of mobile phone technologies to provide medication reminders and further engage PLWH in care has increased [8-11], little is known about the implementation of pre-existing mHealth tools in routine HIV treatment and prevention beyond the initial development or pilot stages.

No previous studies have explored the merits of a widely commercially available predeveloped app to lay the foundation for wide-scale implementation of this evidence-based practice. Thus, within the field of HIV medication adherence, there is a great need to expand beyond the translational, iterative T1 and T2 developmental app stage focused on subgroups of PLWH and into broader implementation of predeveloped mobile health (mHealth) apps such as in a state-wide initiative that is inclusive of a diverse and potentially older population.

mHealth can best be thought of as modern mobile phone platforms that facilitate health interventions through a variety of mobile phone apps. Mechanisms for increasing adherence to mHealth platforms include not only smartphone apps but also interactive voice response reminders and short message service (SMS; eg, digital text messages) [12-20]. Much of the previous work has focused on the development and acceptability of mHealth platforms from the patient’s perspective for HIV care linkage and prevention using both iterative models and the information systems research framework [10,13,16,18-24], mostly with at-risk populations such as men who have sex with men (MSM) [25,26] or youth [27,28]. There is scant literature on the acceptability of a ready-made, commercially available mHealth platform for addressing ART adherence. Further, there are scant data on PLWH aged above 55 years who may not be as likely to own smartphones and may have limitations to data and internet access [29,30]. Therefore, today’s inclusive mHealth platforms also need to include SMS messaging (texting) in partnership with apps that require internet access in order to reach vulnerable populations, known as “biprogrammatic”; we define this term as an app program that functions with both smartphone and texting-only (ie, feature or “flip” phone) platforms. Little is currently known about biprogrammatic platforms and how SMS text interventions are useful in this paradigm, as results have been previously mixed [8,11,15,31-42]. To our knowledge, no study has offered a previously developed, free-of-charge, commercially available mHealth platform that seeks to improve both ART (HIV specific) and other medication adherence (ie, diabetes, mental health, or blood pressure medications) among diverse PLWH with multiple health comorbidities.

## Methods

### Overview

The Florida mHealth Adherence Project for PLWH (FL-mAPP) is an innovative, interventional, clinical pilot study to determine the acceptability and uptake of a mobile phone mHealth intervention platform that incorporates biprogrammatic formats (ie, smartphone and SMS messaging platforms) among PLWH in public health HIV care clinics. More specifically, the study aimed to identify the willingness of PLWH enrolled in public health HIV specialty care clinics to try an existing biprogrammatic mHealth platform called Care4Today [43]; to compare characteristics between self-selecting mHealth users and nonusers, including differences in demographic characteristics, mobile phone type, baseline ART adherence, HIV VL suppression, and attitudes and beliefs; and to identify PLWH preferences for different types of future mHealth interventions.

### Study Design

This study was a cross-sectional analysis of baseline data from a longitudinal mHealth study that offered an ART adherence mHealth intervention and obtained data at baseline (day 0), interim (day 30), and final (day 90) follow-up from self-reported survey responses and queried mHealth app responses. This paper describes baseline and initial update data only, with interim and final data reported elsewhere [44].

### Recruitment

A convenience sample of PLWH in Florida was recruited from three HIV longitudinal care clinics in the Florida Department of Health from 2015 to 2016. Persons eligible to participate in the study were current patients at one of the participating public health clinics that presented within a 4-week window of a scheduled HIV VL test per usual care. Participant inclusion criteria were as follows: confirmed HIV-positive status, on currently prescribed ART, age≥18 years, fluency in written and spoken English, and owning an individual smartphone (eg, iPhone or Android) or feature phone that was able to receive and send text messages. Participant exclusion criteria were as follows: sharing their phone with another person, unwilling or unable to afford any potential fees for receiving and replying to text messages, or visually impaired to the extent that precluded use of the mHealth platform.

Participant recruitment strategies at the three clinics included print flyers in the lobby and clinic waiting areas as well as targeted preclinic visit screening notices to providers for patients who qualified by chart review. Eligible participants were referred by the provider to research staff and then offered an opportunity to participate with signed informed consent.

Both the Institutional Review Boards of the Florida Department of Health and the University of Florida approved the study. Participants were provided a US $10 incentive at baseline and at each of two follow-up intervals for a maximum of US $30 over the study duration.

### Data Collection

After enrollment, all participants completed an initial paper-based survey of 49 questions to capture the demographic, behavioral, clinical, and attitudinal characteristics of PLWH regarding mHealth interventions, informed by previous research on feasibility and acceptability of adopting technology [45,46]. Question items included those on baseline demographics, general items, substance use (ie, tobacco, alcohol, injection drug use, cocaine, heroin, and methamphetamine), and baseline Patient Health Questionnaire-2 screening (to assess comorbid mental health barriers to adherence, and current pill burden). The survey also queried current cellular phone use, current mHealth app use, and attitudes toward the perceived use and utility of mHealth apps for various health-related issues. Specifically, participants were asked to what extent one agreed about the general use of mHealth interventions to manage health and respond using a 5-point Likert scale. The response options of “agree” or “strongly agree” options were combined into one category “agree,” whereas the “neutral,” “disagree,” and “strongly disagree” options were combined into the category “disagree” for statistical facilitation to fit the multivariate model. Participants were also asked how often one would use a free phone app to track alcohol and drug use behavior; communicate with a doctor or clinic; remember to take medication; engage in social networking with other people who live with HIV; connect with family about medications; and track exercise, diet, and weight. Self-reported ART adherence at baseline, 30 days, and 90 days was measured by a follow-up 13-question paper survey item and compared to the corresponding patient self-recorded mobile app responses. ART adherence data available through the app were later presented to the medical provider at the 90-day mark to evaluate if they were consistent with the HIV VLs. Patients were considered adherent to ART if they reported missing less than 2 days of doses in the previous month.

After completion of the baseline survey, research assistants provided information about a specific, existing, commercially available mHealth platform designed to improve medication adherence for multiple health conditions including HIV (Care4Today Mobile Health Manager, Johnson & Johnson, New Brunswick, NJ) [43]. This mHealth interface, biprogrammatic with smartphone (iPhone or Android) and text messaging SMS interfaces, was previously developed commercially and catalogs preprogrammed visual, written, and photographed descriptions of nearly any available medication in the United States to be selected by the user. Once registered, the app cues the participant with visual and optional sound reminders at the selected time of day when the medication is due. The app also records patients’ self-entered dosage completion logs. Such logs are securely stored and available for future retrieval and review by both patients and providers with the patient's consent. The research team kept close logs of participants and was able to download reports directly from the app with patient consent. Participants could also choose to use a more limited mHealth SMS text intervention that would just send written reminders at the time that ART was due, to which the patient could reply with a text message stating “1” to indicate medication completion.

**Figure 1:**
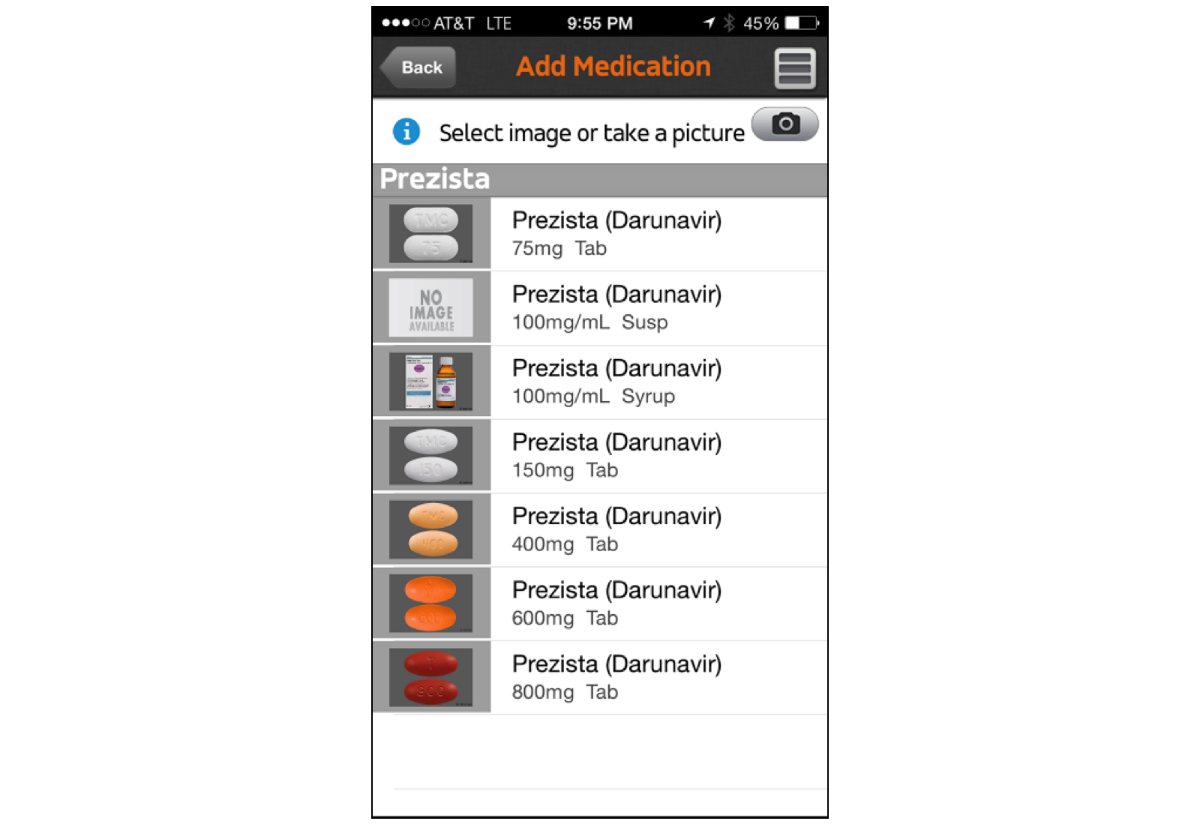
Representative user-facing image for the mobile health app visual for antiretroviral medications as seen in the smartphone program (source: Care4Today Mobile Health Manager, Johnson & Johnson, New Brunswick, NJ).

**Figure 2:**
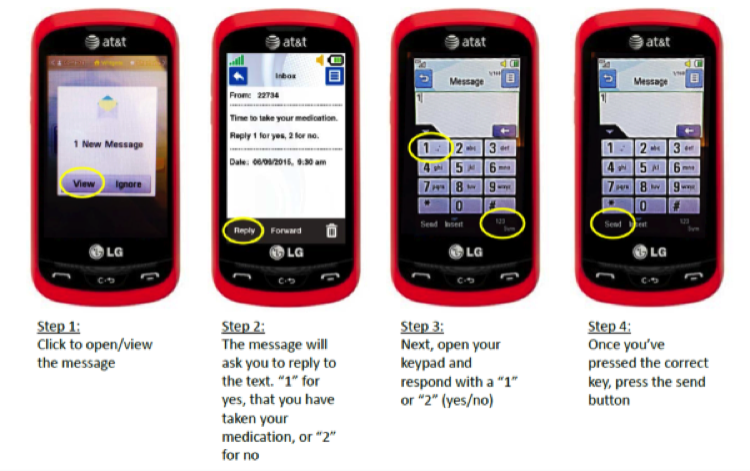
User-facing mobile health app instructions for the antiretroviral app reminder system (source: Care4Today Mobile Health Manager, Johnson & Johnson, New Brunswick, NJ).

No additional monetary incentive was provided above baseline for participants who agreed to use or decline this mHealth platform. Participants self-selected one of three groups: no mHealth intervention, SMS text reminders, or smartphone app reminders. Research assistants helped participants create their accounts and download and configure the app (Figures 1 and 2).

For those who agreed to try the mHealth intervention, the research team tracked whether the participants ever used (responded to text or entered adherence information) the mHealth intervention. For this analysis, “mHealth users” were defined as participants who had at least one recorded interaction with the mHealth platform. *“* mHealth non-users” were defined as those who either declined to try the mHealth platform or agreed to try but never accessed the mHealth platform after enrollment.

Baseline HIV VL level and CD4+ T-cell count were recorded as the most recent value recorded within 30 days of enrollment and were logged at day 30 (short term) and day 90 (long term). HIV viral suppression was defined as <200 copies/mL, per the current US Centers for Disease Control and Prevention, Division of HIV/AIDS, epidemiological definition [2].

### Statistical Analysis

We analyzed baseline data on mHealth user demographics to understand attitudes, beliefs, and willingness to use a biprogrammatic mHealth tool among PLWH in a public health clinic setting. Sociodemographic and attitudinal variables among mHealth users and nonusers were compared using Chi-square tests, with statistical significance set at *P*<.05. A multivariable logistic regression analysis (ie, multivariate analysis) was conducted to identify independent factors associated with successful use of the mHealth platform, as the outcome was binary. Any variables associated with app use in the bivariate analysis with *P*<.20 was included in the multivariable model to maintain inclusivity of variables in a small sample size in a nonimputed model. Missing data were rare, as evidenced in the table format. Results of overall interest in mHealth and preferences for specific types of mobile apps are presented descriptively. All analyses were performed in SAS software, version 9.4 [47].

## Results

### Participants

**Figure 3:**
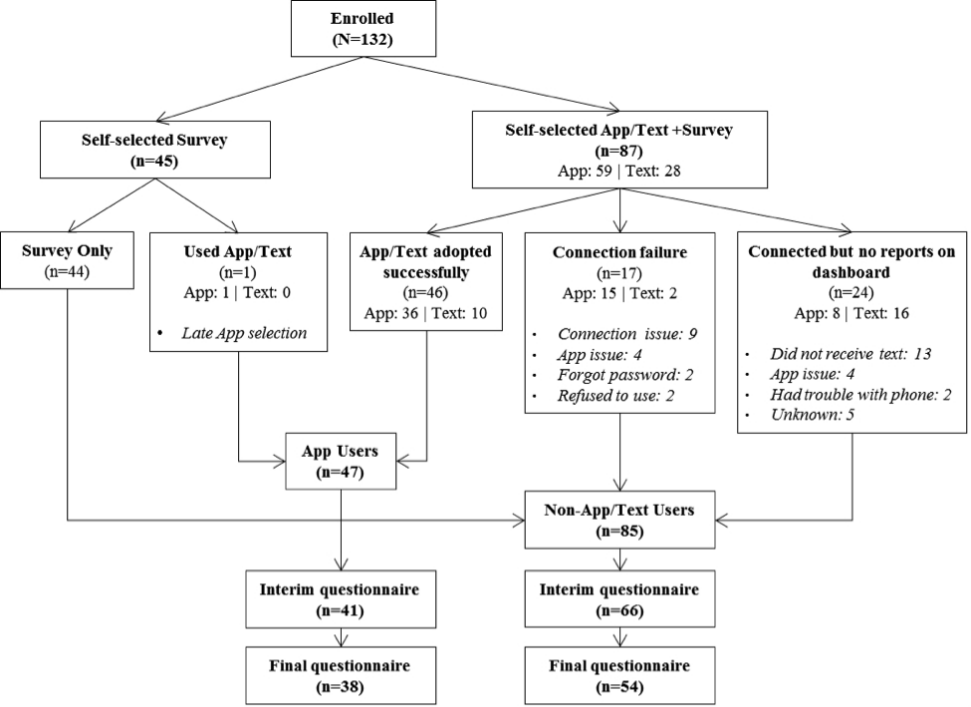
Initial uptake and retention of mobile health users in the Florida mHealth Adherence Project for people living with HIV, 2015-2016 (N=132).

Among the 132 PLWH enrolled, 66% (n=87) initially agreed to use the mHealth platform, of which 54% (n=47) successfully connected to the platform at least once (Figure 3). Nearly 34% of the sample (n=45) self-selected the survey only and did not state a desire to try the app. Unsuccessful attempts to use the app were due to either a reported connection failure (n=17, 20%) or inability or unwillingness to input personal data on adherence (n=24, 28%). Of the 87 PLWH agreeing to use the mHealth platform, we found an approximate 2:1 ratio of persons agreeing to try the smartphone app (n=59) versus the SMS text messages (n=28). Reanalysis of the data into three categories to identify participants who initially had agreed to use the app but who did not ultimately access the app showed no statistical difference in terms of demographics or other covariables: app users (n=45), nonusers (n=43), and those who initially agreed to use the app but ultimately did not use it (n=38). The refusal rate to participate in the study was estimated to be approximately 30% clinic wide and was thought to be mainly due to language barrier or anticipated difficulty with study requirements and not adverse feelings toward technology. However, interested participants who were ultimately referred for a face-to-face visit with the study team had a refusal rate of <5%, likely due to the positive dynamic and cultivated trust between providers, research staff, and clinic patients as well as the noninvasive nature of the study. One participant who had initially declined to use any mHealth option subsequently requested to use the mHealth platform and was reassigned to the mHealth user group, likely due to renewed interest in the app at a later date after the initial educational session.

### User Demographics

Baseline demographics of the sample size of 132 participants were characterized as follows: A majority were men (n=88, 66.7%), were of African American ethnicity (n=79, 60.3%), completed a high school education (n=55, 41.7%), were single/never married (n=88, 66.7%), and were ≥50 years of age (n=58, 46.0%). The average age was 45.8 years (range: 22-69 years). Gender identity, including transgendered identity, but not sexual orientation, was recorded, which is a limitation discussed below.

The majority of participants had a suppressed baseline HIV VL (n=91, 71.0%), with a CD4+ T-cell count > 500 cells/µL (n=69, 54.3%), although by survey, 44% of participants reported <95% ART adherence in the previous month and 28% had detectable HIV viremia at the last laboratory draw.

Importantly, mHealth users were significantly more likely than non-mHealth users to have higher than high school education (57.5% versus 32.9%, *P*=.02) and to already own a smartphone (87.2% versus 67.9%, *P*=.01). However, there were no significant differences in app users and nonusers in terms of age, gender, race, marital status, self-reported ART adherence, HIV viral suppression, and CD4+ T-cell count (Table 1).

### Evaluation Outcomes: Attitudes and Beliefs

Attitudes toward mHealth app uptake at baseline were significantly correlated with the response “having the need to use the app” (*P*=.002), which was defined as the patient’s perception of having difficulty with medication adherence and needing additional support, and the perception of receiving assistance with app navigation (*P*=.04). Peer input or influence, receiving timely medication reminders, and the user-friendly aspect of the app were not significant at baseline for this study group (Table 2).

A multivariate analysis revealed four demographic factors that were significantly correlated with mHealth use: having higher than high school education (adjusted odds ratio [AOR] 2.65; *P*=.05), having confidence that a clinical staff member would assist with mHealth app use (AOR 2.92, *P*=.048), belief that PLWH would use such an mHealth app (AOR 2.89; *P*=.02), and ownership of a smart phone in contrast to a “flip-phone” model (AOR 2.80; *P*=.05; Table 3).

**Table 1:** Baseline characteristics of mobile health platform users and nonusers among people living with HIV/AIDS (PLWH) in the Florida mHealth Adherence Project for PLWH Study, 2015-2016.

Characteristics		Total^a^ (N=132)	mHealth^b^ users (n=47)	mHealth nonusers (n=85)	*P* value
**Age (years), mean (SD)**					.43
	18-29	14 (11.1)	3 (6.7)	11 (13.6)	
	30-39	24 (19.0)	11 (24.4)	13 (16.0)	
	40-49	30 (23.8)	12 (26.7)	18 (22.2)	
	≥50	58 (46.0)	19 (42.2)	39 (48.1)	
**Gender at birth**					.37
	Male	88 (66.7)	29 (61.7)	59 (69.4)	
	Female	44 (33.3)	18 (38.3)	26 (30.6)	
**Race/ethnicity**					.15
	Hispanic	23 (17.6)	13 (27.7)	10 (11.9)	
	White, non-Hispanic	23 (17.6)	8 (17.0)	15 (17.6)	
	Black, non-Hispanic	79 (60.3)	24 (51.1)	55 (65.5)	
	Other, non-Hispanic	6 (4.6)	2 (4.3)	4 (4.8)	
**Education**					.02^c^
	Less than high school	41 (31.1)	11 (23.4)	30 (35.3)	
	High school graduate or GED^d^	36 (27.3)	9 (19.1)	27 (31.8)	
	Higher than high school	55 (41.7)	27 (57.5)	28 (32.9)	
**Marital status**					.11
	Single/never married	88 (66.7)	26 (55.3)	62 (72.9)	
	Divorced/widowed/separated	26 (19.7)	13 (27.7)	13 (15.3)	
	Married/living with a long-term partner	18 (13.6)	8 (17.0)	10 (11.8)	
**Adherence to antiretroviral therapy**					.70
	<95%	57 (44.5)	19 (42.2)	38 (45.8)	
	≥95%	71 (55.5)	26 (57.8)	45 (54.2)	
**HIV viral load suppression (≤200 copies/mL)**					.15
	Yes	91 (71.0)	37 (78.7)	54 (66.7)	
	No	37 (28.9)	10 (21.3)	27 (33.3)	
**CD4+ T-cell count (cells/µL)**					.94
	0-200	15 (11.8)	6 (13.0)	9 (11.1)	
	201-500	43 (33.9)	15 (32.6)	28 (34.6)	
	>500	69 (54.3)	25 (54.4)	44 (54.3)	
**Ownership of a smart phone**					.01^c^
	Yes	98 (74.8)	41 (87.2)	57 (67.9)	
	No	33 (25.2)	6 (12.8)	27 (32.1)	

^a^N does not always total to 132 due to missing data for some items.

^b^mHealth: mobile health.

^c^*P* values are significant (<.05).

^d^GED: General Educational Development.

**Table 2:** Participants’ attitudes at baseline, regarding utility of a mobile health app for people living with HIV/AIDS (PLWH) in the Florida mHealth Adherence Project for PLWH Study, 2015-2016.

Attitudinal items		Total^a^ (N=132)	mHealth^b^ users (n=47)	mHealth nonusers (n=85)	*P* value	
**Mobile app could help me remember to take my medications**						.15
	Agree	112 (85.5)	43 (91.5)	69 (82.1)		
	Not agree	19 (14.5)	4 (8.5)	15 (17.9)		
**Too much effort to learn to use a new app**						.17
	Agree	25 (19.1)	6 (12.8)	19 (22.6)		
	Not agree	106 (80.9)	41 (87.2)	65 (77.4)		
**Most of my friends would agree that I should try a mobile app for medication reminders**						.34
	Agree	68 (51.9)	27 (57.4)	41 (48.8)		
	Not agree	63 (48.1)	20 (42.6)	43 (51.2)		
**Someone at the clinic will help to set up the app if I have difficulties**						.04^c^
	Agree	101 (77.1)	41 (87.2)	60 (71.4)		
	Not agree	30 (22.9)	6 (12.8)	24 (28.6)		
**The app must be fun to use**						.52
	Agree	69 (52.7)	23 (48.9)	46 (54.8)		
	Not agree	62 (47.3)	24 (51.1)	38 (45.2)		
**Will use app only if it is free of charge**						.11
	Agree	89 (67.9)	36 (76.6)	53 (63.1)		
	Not agree	42 (32.1)	11 (23.4)	31 (36.9)		
**I feel like I will need to use the app**						.002^c^
	Agree	76 (58.5)	36 (76.6)	40 (48.2)		
	Not agree	54 (41.5)	11 (23.4)	43 (51.8)		

^a^N does not always total to 132 due to missing data for some items.

^b^mHealth: mobile health.

^c^*P* values are significant (<.05).

**Table 3:** Multivariate correlations between baseline characteristics and adoption of the mobile health platform to improve antiretroviral therapy adherence among people living with HIV/AIDS (PLWH) by multivariable analysis in the Florida mHealth Adherence Project for PLWH Study, 2015-2016 (N=132).

Characteristics		Unadjusted odds ratio (95% CI)	*P* value	Adjusted odds ratio (95% CI)	*P* value
**Education (ref: less than high school)**					
	High school graduate or GED^a^	0.91 (0.33-2.53)	.86	0.85 (0.29-2.52)	.76
	Higher than high school	2.63 (1.1-6.28)	.03	2.65 (1.02-6.86)	.045^b^
**Ownership of a smart phone** **(ref: no)**					
	Yes	3.24 (1.23-8.55)	.02	2.80 (1.00-7.84)	.05^b^
**Someone at the clinic will help to set up the app if I have difficulties** **(ref: disagree)**					
	Agree	2.73 (1.03-7.27)	.04	2.92 (1.01-8.41)	.048^b^
**I feel like I will need to use the app** **(ref: disagree)**					
	Agree	3.52 (1.58-7.84)	.002	2.89 (1.23-6.81)	.02^b^

^a^GED: General Educational Development.

^b^*P* values are significant (<.05).

### Interest in Future Mobile Health Tools

Use of an mHealth app for potentially tracking alcohol or drug use received support from 34.8% (n=16) of mHealth users reporting a theoretical “daily” interest and 68.2% (n=58) of non-mHealth users reporting no interest in such an app (*P*=.002). Overall, 18.3% (n=24) of the study sample expressed occasional interest (Table 4). Interestingly, 70.2% (n=92) reported daily interest in receiving medication adherence reminders via the app (80.4% versus 64.7%), although the value was not significantly different among the user groups (*P*=.06; Table 4).

Attitudes toward social networking with other PLWH were mixed, with overall responses indicating “never/rarely” for this function (n=53, 40.8%) and with nonusers decidedly against such an option (49.4% nonusers versus 24.4% users; *P*=.02).

**Table 4:** Baseline behavioral intent regarding use of a mobile health platform to enhance other forms of health promotion among people living with HIV/AIDS (PLWH) enrolled in the Florida mHealth Adherence Project for PLWH Study, 2015-2016 (N=132). The responses are to the question, “If available and free, how often would you use a phone app to help you?”

Intention item		Total^a^ (N=132)	mHealth users (n=47)	mHealth nonusers (n=85)	*P* value	
**Track changes in mood and emotions**						.45
	Never/rarely	45 (34.6)	16 (35.6)	29 (34.2)		
	Occasionally	36 (27.7)	15 (33.3)	21 (24.7)		
	Daily	49 (37.7)	14 (31.1)	35 (41.2)		
**Improve health by providing tips**						.72
	Never/rarely	22 (16.9)	7 (15.2)	15 (17.9)		
	Occasionally	52 (40.0)	17 (37.0)	35 (41.7)		
	Daily	56 (43.1)	22 (47.8)	34 (40.5)		
**Track alcohol or drug use behavior**						.002^b^
	Never/rarely	75 (57.3)	17 (37.0)	58 (68.2)		
	Occasionally	24 (18.3)	13 (28.3)	11 (12.9)		
	Daily	32 (24.4)	16 (34.8)	16 (18.8)		
**Communicate with doctor or clinic**						.80
	Never/rarely	35 (26.9)	13 (28.3)	22 (26.2)		
	Occasionally	53 (40.8)	17 (37.0)	36 (42.9)		
	Daily	42 (32.3)	16 (34.8)	26 (31.0)		
**Remember to take medication**						.06
	Never/rarely	26 (19.8)	4 (8.7)	22 (25.9)		
	Occasionally	13 (9.9)	5 (10.9)	8 (9.4)		
	Daily	92 (70.2)	37 (80.4)	55 (64.7)		
**Engage in social networking with other people who live with HIV**						.02^b^
	Never/rarely	53 (40.8)	11 (24.4)	42 (49.4)		
	Occasionally	38 (29.2)	17 (37.8)	21 (24.7)		
	Daily	39 (30.0)	17 (37.8)	22 (25.9)		
**Connect with family about medications**						.62
	Never/rarely	83 (63.4)	27 (58.7)	56 (65.9)		
	Occasionally	23 (17.6)	10 (21.7)	13 (15.3)		
	Daily	25 (19.1)	9 (19.6)	16 (18.8)		
**Track exercise, diet, or weight**						.11
	Never/rarely	35 (26.7)	8 (17.4)	27 (31.8)		
	Occasionally	37 (28.2)	12 (26.1)	25 (29.4)		
	Daily	59 (45.0)	26 (56.5)	33 (38.8)		

^a^N does not always total to 132 due to missing data for some items.

^b^*P* values are significant (<.05).

Interest in mHealth tools for tracking changes in mood/emotion, providing health tips, connecting with family, or tracking changes in exercise/diet were mixed and not significant between users and nonusers of the app (Table 4).

## Discussion

### Principal Results

In this study of Florida PLWH recruited from public health clinics, we found that approximately one-third of the persons who were counseled about the opportunity to try an existing mHealth app platform were successful baseline users. mHealth users were significantly more likely to have higher than high school education and own a smart phone. However, successful mHealth use was not significantly associated with age, sex at birth, ethnicity, marital status, baseline ART adherence, baseline HIV VL suppression status, or baseline CD4+ T-cell count, perhaps because these were similarly distributed at baseline.

PLWH baseline attitudes most strongly associated with mHealth use were related to the perceived need to use such an app and perceptions that someone in the clinic would assist them. This finding shows that self-motivation and clinical staff influence can encourage and maintain use of adherence reminders. Even with an older cohort than that described in the literature, age did not make a difference, as expected, probably because of the biprogrammatic interface that allowed for flip phone text use. This has important implications in future mHealth app development, as it highlights the importance of including technical advances for older adults. The biprogrammatic aspect of incorporating SMS/text messaging importantly captured additional participants; if this factor made any clinical impact, further analysis will be needed. In the context of previous studies, these findings are important and inclusive of a diverse cohort of PLWH by age, self-identification, and demographics, whereas previous literature has focused on app development and uptake among specific groups of persons (ie, MSM or youth) and often resulted in “home grown” apps that are not available on a wider or reproducible scale [25,27,28]. One inclusive and robust pharmacological app adherence study by Davies et al [48] was neither HIV specific nor US based. Further, a commercially available app has advantages with regard to sustainability, as it is widely available, consistent across users, and technologically supported for upgrades and security functions as long as privacy options are understood and maintained by both the user and administrator. These findings have important ramifications for implementation science in mHealth, as this successful state-wide pilot study is now ready for scale-up among demographically diverse PLWH [49,50].

Nearly 70.2% (n=92) of participants indicated interest in using an app daily to monitor their medication adherence, if one was available and free; this is promising for an integrative medication reminder model among more educated and mobile phone–savvy PLWH. However, more research is needed to maintain interest and trust among app users and to make apps relevant to care for both the patient and provider. Integrating positive feedback such as suppressed HIV VLs and other imported positive lab results into the app may increase both app and medication adherence over the long term.

A strength of this study is that it included a diverse group of PLWH across all demographics, ages, and risk factors; future studies should incorporate a wider geographical expanse of clinics using the idea of a biprogrammatic, free-of-charge, commercially available app to further refine an app that would be useful for the widest array of PLWH, across all health conditions, and appropriate to an aging PLWH population that often has the highest pill burden.

### Limitations

This study had a few limitations that need to be addressed. First, participants were recruited only within public health clinics; however, this was designed to be a pilot study, and the results should be generalizable to PLWH who are seen in public health clinical settings. The participating public health clinics captured a wide variety of sociodemographic groups and can be considered representative of the Ryan White Funded HIV clinics in the state of Florida [51]. Although the exact income levels were not recorded, individuals receiving Ryan White support met certain income requirements. Second, the survey and app only included English-speaking individuals; more interventions should be considered for Spanish-speaking and Haitian Creole–speaking populations, especially in states such as Florida. Third, the current report only considers factors associated with baseline characteristics, general interest in mHealth tools, and initial use of the mHealth tool. Collection of information on the sexual orientation, and not just gender identity, would have been useful for this cohort of participants, given the extensive prior work among MSM youth in the literature, especially since mHealth pilot interventions for HIV have mainly focused on this risk group.

### Comparison with Prior Work

Prior work on mHealth apps for PLWH has focused on the *de novo* development or iteration of an app or new technology in a subset of PLWH (ie, grouped by age or risk factor status). In contrast, this study focuses on the baseline and long-term usability of a ready-made, freely available, commercial app for ART adherence among a diverse population of PLWH in a biprogrammatic platform. This would be relevant and important in a larger, national scale-up for a translational technological link between the patient, app, and provider to synchronize medical adherence to virological suppression and thus to real-time clinical outcome success. This commercially available app is free of charge and updated regularly to ensure security and usability.

### Conclusions

The FL-mAPP successfully demonstrated uptake of a widely available, free-of-charge, commercially available mHealth medication adherence app in a diverse population of PLWH. The results are promising, as they illustrate the usefulness of a biprogrammatic platform inclusive of diverse and aging PLWH users. For research on HIV implementation science focusing on mHealth, this successful statewide pilot study can be implemented on a larger scale.

## Abbreviations

AOR: adjusted odds ratio
ART: antiretroviral
FL-mAPP: Florida mHealth Adherence Project for PLWH
GED: General Educational Development
mHealth: mobile health
MSM: men who have sex with men
PLWH: people living with HIV
SMS: short message service
VL: viral load
